# Anosmia Testing as Early Detection of SARS-CoV-2 Positivity; A Prospective Study under Screening Conditions

**DOI:** 10.3390/life12070968

**Published:** 2022-06-28

**Authors:** Frederic Jungbauer, Catharina Gerhards, Margot Thiaucourt, Michael Behnes, Nicole Rotter, Angela Schell, Verena Haselmann, Michael Neumaier, Maximilian Kittel

**Affiliations:** 1 Department for Otorhinolaryngology, Head- and Neck-Surgery, University Medical Centre Mannheim, Theodor-Kutzer-Ufer 1-3, 68167 Mannheim, Germany; frederic.jungbauer@umm.de (F.J.); nicole.rotter@umm.de (N.R.); angela.schell@umm.de (A.S.); 2 Institute for Clinical Chemistry, Medical Faculty Mannheim of the University of Heidelberg, Theodor-Kutzer-Ufer 1-3, 68167 Mannheim, Germany; catharina.gerhards@umm.de (C.G.); margotte.t@gmail.com (M.T.); verena.haselmann@umm.de (V.H.); michael.neumaier@umm.de (M.N.); 3 German Center for Cardiovascular Research, First Department of Medicine, Faculty of Medicine Mannheim, University Medical Centre Mannheim (UMM), University of Heidelberg, DZHK, Partner Site Heidelberg/Mannheim, Theodor-Kutzer-Ufer 1-3, 68167 Mannheim, Germany; michael.behnes@umm.de

**Keywords:** COVID-19, anosmia, case-control-study, screening-test, olfactory dysfunction, prediction model, Boruta algorithm

## Abstract

Sudden onset of anosmia is a phenomenon often associated with developing COVID-19 disease and has even been described as an initial isolated symptom in individual cases. In this case-control study, we investigated the feasibility of this condition as a suitable screening test in a population at risk. We performed a prospective study with a total of 313 subjects with suspected SARS-CoV-2 infection. In parallel to routine PCR analysis, a modified commercial scent test was performed to objectify the presence of potential anosmia as a predictor of SARS-CoV-2 positivity. Furthermore, a structured interview assessment of the participants was conducted. A total of 12.1% of the study participants had molecular genetic detection of SARS-CoV-2 infection in the nasopharyngeal swab. It could be demonstrated that these subjects had a significantly weaker olfactory identification performance of the scents. Further analysis of the collected data from the scent test and medical history via random forest (Boruta) algorithm showed that no improvement of the prediction power was achieved by this design. The assay investigated in this study may be suitable for screening general olfactory function. For the screening of COVID-19, it seems to be affected by too many external and internal biases and requires too elaborate and selective pre-test screening.

## 1. Introduction

In the context of the worldwide pandemic caused by the novel coronavirus Severe Acute Respiratory Syndrome Corona Virus 2 (SARS-CoV-2), the infectious disease Corona Virus Disease 2019 (COVID-19) leads in the most severe cases to acute respiratory failure with the need for invasive ventilation and intensive care [1]. At the same time, however, a large proportion of cases of COVID-19 present with much milder, sometimes oligo- or asymptomatic courses, with only cough, fever, and other mild flu-like symptoms that are self-limiting [2].

As a strikingly specific symptom, acute hyposmia and anosmia were observed early in the course of the disease, especially in the case of infection with the wild type of the virus (2019-nCoV; HCoV-19) or the alpha variant (B.1.1.7). In this case, reductions in the sense of scent already occur a few days after the infection and persist parallel to the infectious course of the disease [3].

The underlying pathomechamisms are not yet completely understood. In conventional upper respiratory tract infections, such as viral rhinitis, olfactory dysfunction can be explained mainly by mechanical obstruction of the epithelium of the olfactory groove by mucus formation and mucosal swelling. In addition, the mechanism of postviral olfactorial dysfunction is known to occur with a time delay after the classic symptoms during the acute infection, such as rhinorrhea and nasal obstruction, has subsided. Here, a molecular mimicry leads to an autoimmune reaction of antiviral antibodies against the olfactory epithelial cells [4]. Thus, the hyposmia is not the result of the toxic effect of the viruses themselves, but of the misdirected immune response. In contrast, in the case of early olfactory impairment in the context of SARS-CoV-2 infections, the hypothesis is that the symptoms are caused by a direct neurotoxic effect of the virus [5]. Current knowledge suggests that the angiotensin-converting enzyme 2 (ACE2) receptor plays an important role in viral penetration of the nervous system by SARS-CoV-2, since the virus has a high affinity for the receptor that is strongly expressed in the nasal mucosa among other organs. Olfactory symptoms appear to occur relatively early in the course of the disease, while other neurological manifestations, e.g., paralysis of eye muscles and other cranial nerves, occur later, probably associated with additional other pathomechanisms such as vascular inflammation [6].

As a COVID-19 symptom, hyposmia/anosmia is experienced only by the affected person and cannot be easily objectified unlike cough or fever, which can be perceived and communicated by bystanders. There is no external surveillance, which can make patients with isolated olfactory symptoms potential unrecognized super-spreaders. At the same time, the subjective perception of one’s own sense of smell is also highly variable. The well-known physiological adaptation to an initially perceived scent, which in the course of time passes over into the unconsciousness and the habitual inhomogeneity of fragrances in everyday life (e.g., in the case of differently spiced meals) are further reasons why, in the case of objectively detectable hyposmia, a medical condition may not be recognized by the affected person and consultation would not primarily follow.

Because of this special status, the query for olfactory changes during the COVID-19 pandemic was used as a screening query to capture oligosymptomatic courses in addition to the classic symptoms of infection (fever, cough, general feeling of illness) [7].

With increasing test capacity and higher vaccination rates, sporting, cultural, and social events now recommence subject to appropriate hygiene guidance. For population-wide testing, rapid SARS-CoV-2 antigen tests are a key-component. While their advantage lies in their rapid availability, ease of use, and result evaluation, they are inferior to thesensitivity and specificity of polymerase chain reaction (PCR) tests considered the current gold standard in COVID-19 diagnostics. Both false-positive and false-negative results may occur [8]. Therefore, to improve the predictive values of these tests, it is critical to increase the pre-test probability. This can be achieved using test strategies that will step-wise increase prevalence of the condition in question and thus improve positive predictive values of test results.

Accordingly, an objectifiable test of the olfactory sense is a strong candidate for the identification of otherwise oligo- or subjectively asymptomatic individuals and may be efficient to target affected individuals for further testing. Complex olfactory testing is often carried out in hospital settings in the context of scientific studies. Specifically, testing by olfactometer as the gold standard is primarily reserved for studies due to the high technical effort involved. The clinical standard for scenting evaluation usually includes so-called Sniffin’ sticks [9], which are used to test the threshold (T), discrimination (D), and identification (I) of odors. Normosmia, hyposmia, or anosmia are then evaluated based on the TDI score. In contrast, an uncomplicated instrument for broad application in screening scenarios has not yet been established.

In order to validate the value of so-called scratch tests, the combination of anamnesis by standardized questionnaire, subsequent olfactory testing by a scratch test, and PCR testing via standard nasopharyngeal swab was investigated in a prospective study in order to evaluate the clinical utility of a smelling test as a first-level entry into a step-wise screening strategy.

## 2. Materials and Methods

### 2.1. Study Design and Setting/Patient Recruitment

The study was conducted as double-blinded observation study for 6 weeks between May and July of 2021 at a university hospital center in South-West Germany. Participants visiting the outpatient SARS-CoV-2 PCR-testing center included in the study on a voluntary basis following written consent. The study was approved by the local ethics committee (registration number: 2020-556 N). The respective group assignment, case or control, was made according to the molecular genetic test result.

By regulation of the state of Baden-Württemberg (*Corona Testverordnung*) at that time, public SARS-CoV-2 testing is preconditioned on the following circumstances [10]: (a) non-specific symptoms of a respiratory infection, (b) specific symptoms of COVID-19, such as anosmia, (c) first-degree (unprotected) contact with a confirmed SARS-CoV-2 infected person, and (d) eligibility as healthcare professionals returning from a high-risk area as defined by the German Robert Koch Institute (RKI) [11].

### 2.2. Olfactory Evaluation Card

To assess the participants’ scenting capability, commercially available olfactory evaluation cards (OEC) designed as scratch tests were used (COROWELL, MKG Consulting UG, Bad Neuenahr-Ahrweiler, Germany). This OEC comprises a folded testing card of credit card format unfolding to a size of 5.5 cm by 17 cm. It features a two-dimensional barcode identifier and a scratchable scent-coated test field the size of 2.5 cm by 2.5 cm, each on the inside. Furthermore, a 4-step instruction-of-use information is provided on the inside of the OEC (Supplementary Material S1). For the study, we randomly combined three OEC with different scent-coatings for the participants to use. All OEC used were identified by a sequential barcode.

### 2.3. Study Process, Demographic and Clinical Data Collection

The study design was double blinded, and participants contributing were instructed by the investigators on how to perform the test and which of the 9 potential scents (cherry, pine tree, chocolate, clove, vanilla, spearmint, lavender, orange, and cinnamon) could be perceived. In addition, the participants were given the false information that some scenting field may not feature a scent in order to reduce pressure-to-respond in the case of anosmia and thus reduce a potential response bias. After performing the 3 scent tests, a short, standardized survey was conducted with each individual participant regarding questions on age, subjective assessment of their general scenting ability, smoking behavior, their medical history potentially affecting scenting ability, as well as a positive SARS-CoV-2 test, i.e., confirmed virus infection during the previous 2 months. A detailed overview of the questionnaire can be found in the Supplementary Material S2.

Barcode scanning and data collection was directly transferred into a SQL database using an Android application (scan-IT to Office, Version 4.5.0.33460; TEC-IT Datenverarbeitung GmbH, Steyr, Austria). Reconciliation of the scents smelled and the scent deposited on the OEC was carried out through database query of the manufacturing records (COROWELL, MKG Consulting UG, Bad Neuenahr-Ahrweiler, Germany). To assess the predictive power of the OEC test, the following 4 scenarios were evaluated: (a) only the first ticket was rated; (b) one of 3 scents had to be correctly identified to pass the test; (c) 2 of 3 scents had to be correctly identified; and (d) all scents had to be correctly identified. Since the test participants had to detach their mouth/nose protection to perform the test, the examinations were carried out only in the outside area in wind-protected surroundings.

### 2.4. Molecular Testing

The detection of SARS-CoV-2 was performed in a biosafety class II laboratory under DIN-EN ISO 15189 conditions. Trained healthcare professionals performed a deep nasopharyngeal swab with a medical swab on all participants. No additional transport media were used, and all swabs were resuspended in 2 mL 0.9% sodium chloride solution within 3 h.

The Roche Cobas^TM^ SARS-CoV-2 (Roche, Mannheim, Germany) assay was used for the detection of SARS-CoV-2 viral particles on the 6800 Platform (Roche, Mannheim, Germany). This assay is a double target, quantitative real-time reverse transcriptase polymerase chain reaction (qRT-PCR) amplifying the *ORF1* gene unique to SARS-CoV-2 together with the *E-gene*, a conserved region for pan-Sarbecovirus detection. The assay was carried out according to the manufacturer’s instructions.

Samples were classified positive for SARS-CoV-2, when an amplification product for *ORF1* was obtained. We also classified the test “presumed positive”, if an amplification product was detected for the E-gene, only.

For the subsequent data evaluation, the participants were classified into the respective groups based on their molecular genetic test results.

### 2.5. External Conditions

The weather data, temperature, and humidity, were retrospectively extracted from an online database for the greater Mannheim area [12] to account for external conditions that could potentially influence a successful identification of scents.

### 2.6. Prediction of SARS-CoV-2 PCR Test Positivity Using Olfactory Evaluation as a Screening Tool

Furthermore, we wanted to investigate whether it is possible to predict a SARS-CoV-2 infection by olfactory testing. Due to the test design consisting of 3 consecutive scent tests, different cut-offs were evaluated as a passing limit for the overall test. Furthermore, an evaluation was performed to consider how it would have looked in a single test. In that case, only the correct answer to the first scent field was rated as a pass for the entire test.

### 2.7. Scent Comparison

Besides, the individual scents were compared regarding their discriminatory power. The identified scents of all tickets were grouped according to their molecular genetic test results and analyzed in detail. The significance in these different groups was subsequently assessed.

### 2.8. Statistical Analysis

The study population was characterized using descriptive statistics. The significance of test results was evaluated by Fisher’s exact test for connected samples. *p*-values < 0.05 were rated as statistically significant. All analyses were performed with the open-source program The R-Project^TM^ (R-Core Team, version 4.1.2, sourced from r-project.org). The corresponding components as indicated and with the use of the graphical user interface jamovi^TM^ (The jamovi project, version 1.6, sourced from jamovi.org).

### 2.9. Statistical Analysis—Performance of Feature Selection

In order to identify further potential predictive factors, a feature selection was performed using Boruta algorithm. It is an enhancement of the random forest method that uses the original algorithm’s generated importance measure. This is compared with the importance of randomized copies. We applied a feature selection algorithm to examine the prediction suitability of olfactory tests with respect to a positive SARS-CoV-2 qRT-PCR result from nasopharyngeal swabs. For this purpose, we first used the Boruta algorithm, a random forest approach that generates shadow variables from each real variable via permutation [13,14]. Statistical tests are performed comparing the maximum shadow variable and the real variables. If the maximum importance of the real variable is significantly higher, it is evaluated as highly important by the algorithm. These parameters were further used for a logistic regression with positive SARS-CoV-2-qRT-PCR result as target variable. In a second approach, we wanted to examine the influence of clinical parameters on a successful identification of the scent when tested once. In both approaches, an internal cross-validation was performed with equally sized data sets (training dataset *n* = 156, 16 pos. qRT-PCR, validation dataset *n* = 156, 21 pos. qRT-PCR). Receiver operating characteristics (ROC) analysis was applied for determination of the area under the curve (AUC). Feature selection, logistic regression, and ROC were performed using R statistics software (R-Core Team, Version 4.1.2, sourced from r-project.org).

## 3. Results

### 3.1. Study Population

A total of 313 subjects were recruited for the study on a total of 25 recruitment days, between the 20th of May and the 1st of July 2021, of whom 175 (55.9%) identified themselves as female, the remaining participants identified themselves as male. The median age of the study population was 27 years, ranging from 8 to 77 years (mean: 31.97 years, standard deviation of 14.28). The demographic statistics of SARS-CoV-2 positive participants did not significantly differ to those of the SARS-CoV-2 negative (data not shown). The assessment revealed that a total of 38 subjects (12.10%) had a molecularly detectable infection with SARS-CoV-2 at that time. There was a significant difference regarding gender distribution between the case and control group (Fisher´s exact test *p* = 0.005), while there was no significant difference in age distribution. No statistically significant differences were found between the case and control groups for the attributes hay fever/cold, smoked during the last 2 h, subjective scent ability, and inspired through the nose (Fisher´s exact test *p* = 0.1278; *p* = 0.8055; *p* = 0.6736; *p* = 0.3465; *p* = 0.24). In the case of 5-level semi-quantitative response scales, very good and good were always combined and compared to the lower levels (Table 1 and Supplementary Material S3).

A total of 14 of the case-group participants reported having been tested positive for SARS-CoV-2 within the last two months, while a total of 41 subjects (13.12%) in the whole study population reported that. No distinction was made between antigen and molecular testing regarding this item. The prevalence of the attribute tested positive during the last two months differed significantly in the two groups (Fisher´s exact test *p* = 0.0001) (Supplementary Material S4).

The respective 7-day incidence per 100,000 inhabitants for this area was 15.8 at the time the study was performed (median 16.3, standard deviation 2.48) [15].

### 3.2. External Conditions

The analysis and visual representation of the environmental influences were presented in the form of a mixed correlation matrix using the R-studio plugin corrplot. The spearman correlation serves as the mathematical basis for this method [16].

With the help of the correlation matrix and the diagrams thus generated, which show the correlations between the pairs of variables, the influence of environmental factors in the outside area could be excluded [17]. The correlation between the number of correctly answered OECs and the environmental factors in the control group ranged from −0.21 to 0.2 (Supplementary Material S5).

### 3.3. Evaluation of the Series Measurement

**Figure 1:**
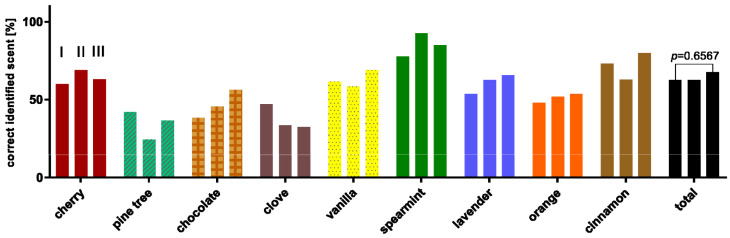
Percentage distribution of correctly identified scents, of the corresponding test rounds (I, II, III). Pine tree and cherry showed a poorer identification rate between the first and the third test approach. All these differences were not significant.

Since olfactory perception is a phasic-tonic sensory stimulus, there would have been a likelihood that the participants would have performed worse due to the sequential study design [18]. For this reason, the correct answer rates of the control group were examined separately according to a scent and test approach. Only pine tree and cherry showed a poorer identification rate between the first and the third test approach as displayed in Figure 1. All these differences were not significant, and thus a bias in the study design could be excluded.

### 3.4. Evaluation of the Test Scenarios and Cut-Off Determination

The study design enables the use of several cut-offs to define the thresholds at which the assay is considered pathological/failed and therefore indicates the presence of SARS-CoV-2 infection. Table 2 illustrates how these different thresholds affect the sensitivity, specificity, and the receptive predictive values. As expected, a high sensitivity of 86.8% could be achieved by a severe cut off (three out of three tickets have to be correctly identified), the corresponding specificity was 25.8%. Based on the current regional prevalence, the positive predictive value obtained was 13.9%, and the negative predictive value was 93.4%. For a low cut off (one out of three tickets have to be correctly identified), a sensitivity of 28.9%, a specificity of 86.5%, and a positive and negative predictive value of 22.9% and 89.8%, respectively, were obtained. The most relevant scenario is represented by the stand-alone evaluation of the first ticket. Hereby, a sensitivity of 68.4% and a specificity of 54.9% were achieved. Considering the prevalence, positive and negative predictive values of 17.3% and 92.6%, respectively, were obtained.

### 3.5. Subgroup Analysis

In order to identify whether the assay was more suitable for a specific set of patients, the authors defined and analyzed six subgroups based on gender and other characteristics. These subgroups were defined: (i) female, (ii) male, (iii) smoked during the last 2 h, (iv) cold/hay fever, (v) scenting sense good and very good, and (vi) tested positive for SARS-CoV-2 during the last two months.

**Figure 2:**
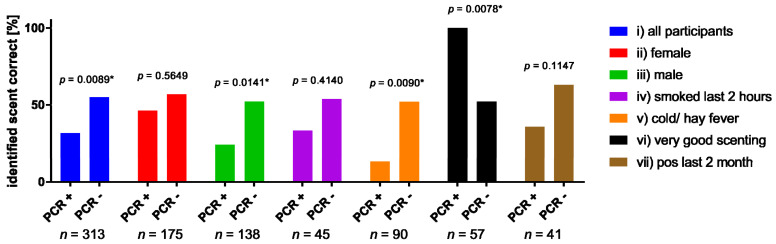
Comparison of identifications performance between subjects with molecular genetic evidence of SARS-CoV-2 and without. In addition to the total study population (**i**), males and females (**ii**,**iii**), as well as smokers (**iv**), participants suffering from allergies or cold (**v**), participants who considered their sense of olfaction to be very good (**vi**) and participants who had already tested positive for SARS-CoV-2 within the last 2 months (**vii**) were investigated separately. (* indicates significance throughout Fisher’s exact test).

It was observed that the identification performance was almost permanently better in the PCR negative/control group. This was only reversed in subgroup (v) scenting sense good and very good. Furthermore, it could be proven that the difference in groups (i), (ii), (v), and (vi) is significant (Fisher´s exact test: (i) *p* = 0.0089; (iii) *p* = 0.0141 (v) *p* = 0.0090; and (vi) *p* = 0.0078). In addition, it is shown that the identification performance of the PCR negative/healthy participants falls considerably under the expected performance and ranges between 62.93% and 52% (9). With regard to the different scent qualities, no differences were found in the individual subgroups (Table 3, Figure 2, and Supplementary Material S6).

### 3.6. Scent Comparison

**Figure 3:**
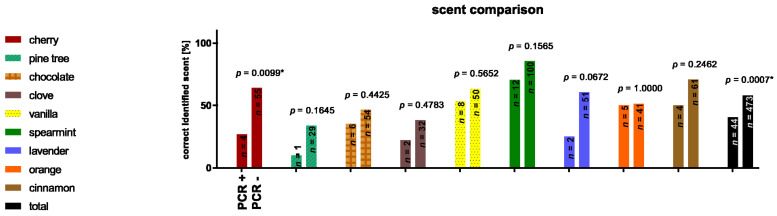
Percentage distribution of correctly identified scents, differentiated by molecular genetic testing result. This shows that the control (PCR negative) group achieves a better identification performance. (* indicates significance throughout Fisher’s exact test).

A total of 517 correctly answered OECs could be analyzed, with 40.4% (*n* = 44) from the PCR positive case group versus the 57.8% (*n* = 473) from the PCR negative control group being correctly identified. Respectively, 9% (*n* = 12) of the case group stated that they had smelled a fragrance but could not identify it or did not smell anything. In the control group, 5.9% (*n* = 53) could not identify the corresponding scent and 2.5% (*n* = 22) stated that they did not smell anything. These differences proved to be significant (data not shown). While the differences in the overall analysis of all scents were significant (*p* = 0.0007), this was only confirmed for the scent cherry (*p* = 0.0099) in the sub-analysis. In both the case and control groups, pine was the scent with the lowest correct identification rate in percentage (10% vs. 33.7%) and peppermint was the scent with the highest identification rate (70.6% vs. 85.5%) (Figure 3).

### 3.7. Supervised Machine Learning Based Feature Selection

**Figure 4:**
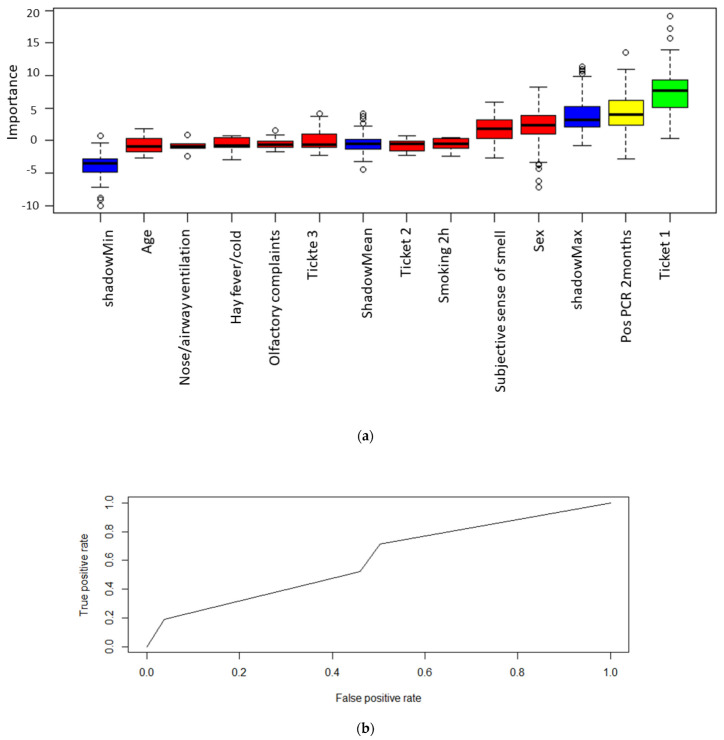
(**a**): A random forest algorithm was used to assess variable importance with regard to predict a positive qPCR result. High importance is illustrated by the color green, medium importance by yellow and low importance by red. Moreover, the minimum, mean and maximum importance of the shadow variables are shown in blue. Parameters with a lower relevance for predicting a positive SARS-CoV-2 qPCR results compared to the maximum shadow variable have been assigned a low importance and thus were not included into the prediction model. (**b**): Logistic regression was performed using the variables “olfactory test” and “positive qPCR before two months” for predicting a positive qPCR results. The true positive rate is compared to the false positive rate and the area under the curve (AUC) is integrated into the illustration.

**Figure 5:**
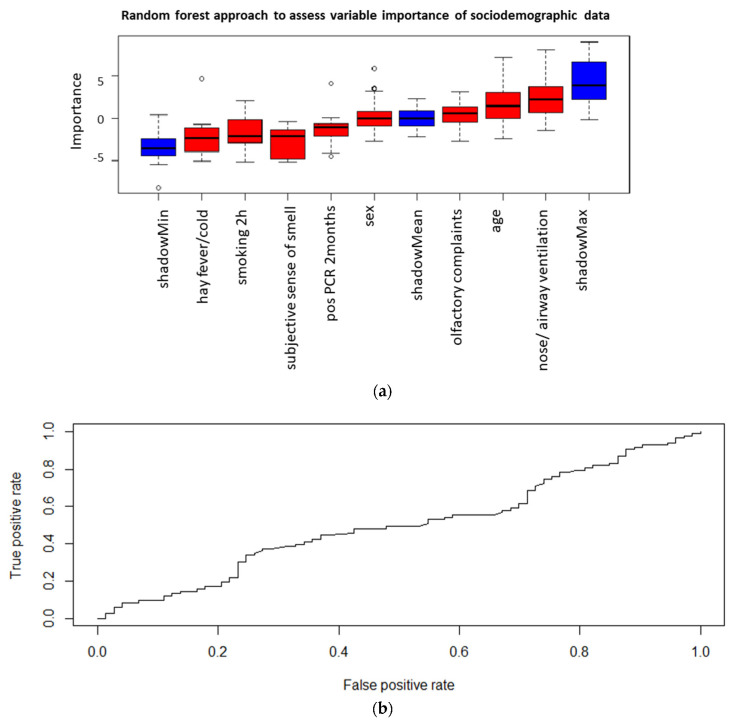
(**a**) The importance to predict a positive olfactory test result was examined. High importance would be illustrated by the color green, medium importance by yellow and low importance by red. Additionally, the minimum, mean and maximum importance of the shadow variables are shown in blue. Parameters with a lower relevance than maximum shadow variable have been assigned a low importance. This analysis was performed to exclude a bias by sociodemographic data. (**b**) Logistic regression was performed using all sociodemographic data for predicting a positive olfactory test results. The true positive rate is compared to the false positive rate and the area under the curve (AUC) is integrated into the illustration.

For predicting a positive qRT-PCR result, the Boruta algorithm assigned high importance to the parameter “ticket_1” and a medium relevance to “positive qRT-PCR result 2 months ago” (Figure 4a). Logistic regression with both parameters was performed using the validation dataset. The results of the ROC analysis are shown in Figure 4b. The AUC of 0.61 was obtained for the prediction of a positive SARS-CoV-2 PCR by scenting tests and previous positive qRT-PCR result. In addition, we examined which clinical parameters influenced the correct identification of scents. Among the parameters age, gender, olfactory complaints, smoking within the last 2 h, subjective sense of smell, hay fever_cold, reduction of nasal ventilation, and positive SARS-CoV-2 qRT-PCR in the last 2 months, the random forest approach rated no parameter with high importance for the correct response of olfactory tests (Figure 5a). The application of all clinical variables in the prediction model showed an AUC of 0.5 (Figure 5b).

## 4. Discussion

In this prospective case-control study, we evaluated the presence of acute olfactory dysfunction as well as its potential as a diagnostic approach under realistic conditions and with a subject population next to routine molecular virus testing.

Early loss of sense of smell and taste is described as a specific syndrome for COVID-19 disease after infection with SARS-CoV-2 virus, in contrast to other nonspecific infection symptoms, such as fever or cough [19]. Because acute loss of the olfactory sense is rare in the healthy (relative to SARS-CoV-2 infection) community [20], its occurrence in the current COVID-19 pandemic must prompt further investigation, such as PCR testing.

Previously, this at least partially specific symptomatology was used as an anamnestic screening tool, along with general symptoms of upper respiratory tract infection, such as cough, fever, and rhinitis. Based on studies showing the strongest association of olfactory impairment with COVID-19 compared with these other symptoms [21,22], commercially available scratch tests have been developed to test the sense of smell in an ambulatory and simple manner and to derive a conclusion about the likelihood of COVID-19 [23]. One such test was investigated in the study presented here and evaluated for its usefulness as a screening method.

It is noteworthy that the scent test performance in terms of identifying SARS-CoV-2 infections is considerably lower than comparably published studies [23,24]. However, it should be mentioned that this study is one of the rare ones where the scent test was performed under screening conditions. This leads to a much lower prevalence in the study population, resulting in reduced positive predictive values, and leading to the detection of very early infections. Since the onset of symptoms of anosmia in similar studies would appear on average after 4.4 days, they might not be identifiable in the study design [25].

The ability to perceive and recognize scents is known to decrease with age [9,26]. However, even in a shorter period of time, the sense of smell is subject to qualitative fluctuations. On the one hand, these are internal factors, such as attention or adaptation to stimuli. At the same time, external influencing factors also determine the ability to smell. It is well known that the sense of smell deteriorates with recent or chronic tobacco use [27]. It also seems likely that (when tested outdoors) wind and turbulence have an influence on the transport of substance particles to olfactory cells. The sense of smell does not appear to be affected by temperature or humidity in healthy young subjects [28]. In contrast, current barometric pressure showed an effect on threshold odor sensitivity in previous studies but not on suprathreshold scent discrimination [29]. In our data, there were no correlations or dependencies of subjects’ test performance with current weather conditions. This indicates that testing is reproducible under altered climatic conditions and that diverse weather conditions do not per se limit the test. However, weather factors are at best measurable outside of a laboratory, but not experimentally controllable, so the validity of this derivation is limited. It is, however, in consensus with the current literature. Nevertheless, even with the seemingly simple olfactory test, there are a number of influencing factors that can falsify the result and thus the significance of the test. After the scent has been perceived by the brain, it must be compared with the already existing olfactory memory. While the triggering of emotions, be they positive or negative, can be done with a subconscious matching of the odor memory, this must be done actively and consciously when searching for the correct name of the scent, if it is not a very familiar and routine scent from everyday life [30]. This requires that the odor to be tested has already been perceived at least once, that in this situation the origin of the odor was known, and that a naming of the odor or its source substance has taken place. This already shows a clear limitation: scents and the ability to name their origin are globally very heterogeneously distributed, e.g., in the case of plants or fruits due to botanical-geographical borders or in the case of spices due to culinary-cultural characteristics. The international establishment of a single test set with the same scents would therefore represent a relevant bias. It seems rather necessary to compile country- or area-specific test sets, just as has been done for other established olfactory tests [31,32,33]. In addition to the identification of the scent and the internal recognition, however, the test investigated here also checks the possibility of external verbalization of the result. A person who recognizes the scent himself, but is unable to verbalize the corresponding name for it due to a language barrier, will get a false-bad test result. Depending on the language and vocabulary competence of the tested group, a significant deviation of the average correct results is therefore to be expected. Our test was conducted in German, but it may be possible that due to a relatively high proportion of foreigners in our sample there was a bias in the average results [34]. This bias could be counteracted by means of various methods, such as presenting the scents available for selection as pictures, but complete neutralization seems difficult. Therefore, subjects with limited language capabilities in the test language would have to be preselected and, if necessary, excluded from the test, as the test result would not be usable.

We introduced the choice option of “did not smell” in our study to identify potential subjective anosmia and to investigate a possible correlation between subjective anosmia and positive SARS-CoV-2 tests. This softened the forced-choice test principle, as subjects were no longer required to select one of the available scents, but were able to revert to the “did not smell” response option in the case of subjective anosmia. On the one hand, this made it possible to identify subjective anosmia, which would not have been possible with real forced-choice, since the subjects would then have guessed and chosen one of the selection scents. On the other hand, this results in a poorer comparability of our results with established test methods, since these are mostly based on the forced-choice principle and accordingly yield different thresholds for the evaluation of hyposmia or anosmia. These include the Sniffin’ sticks with the evaluation of the TDI score [9]. It seems advisable to screen patients for subjective complete anosmia before testing and to exclude it for the scenting test. Subsequently, a forced-choice procedure can be specified for the answer selection and the corresponding reference values of the established scents tests can be adopted.

Even though the screening test investigated here was primarily developed as a single test, it may become necessary to retest, e.g., due to technical problems. Two theoretical factors play a role in a possible change in test performance between the initial test and the retest: On the one hand, it seems logical that a subject could perform better in the retest than in the initial test due to a now more secure understanding of the test procedure. On the other hand, habituation to repeatedly presented scents is a well-known neurobiological phenomenon [35], and therefore a deterioration of test performance could also be possible. However, our results show no significant difference between initial and repeated testing. On the one hand, this is probably due to the simple experimental design, so that no significant comprehension advantage occurs in the retest. At the same time, no relevant habituation occurs due to the variation of the scents offered, so that no disadvantage results from a repeated measurement.

If hypo- or anosmia is detected in the olfactory test, especially if it occurs acutely without accompanying symptoms, such as subjective nasal obstruction or rhinorrhea, it is recommended to perform further SARS-CoV-2 diagnostics, preferably by PCR diagnostics. It should be emphasized that even a negative test for COVID-19 should be followed by further workup of a first-time hypo- or anosmia. In this case, an ear, nose, and throat specialist is recommended for a clean history, clinical examination, and performance of olfactory testing using established methods. On the one hand, this is necessary, because even an olfactory impairment that does not interfere with everyday life can be an early symptom of relevant diseases (such as esthesioneuroblastoma or Parkinson’s disease [36]). On the other hand, the affected person must be adequately informed about this sensory impairment and necessary precautions, such as equipping the home with smoke detectors in the absence of the ability to smell smoke, must be explained.

Other nervous deficits in the context of COVID-19 have been described rather rarely. Although there are case reports of individual motor deficits, such as ocular muscle paralysis [37], facial nerve palsy [38], or recurrent nerve palsy [39], other sensory deficits, such as hearing or balance, have not been observed in clinical studies [40], which again emphasizes the importance of olfactory loss in the context of COVID-19. Against this background, therefore, especially those stimuli that address another perceptual quality in addition to the olfactory sensory system must be scrutinized, such as, more precisely, trigeminal stimulation. One such scent used in the scratch tests examined here is peppermint [41]. On the one hand, the high identification rate of peppermint can be explained by its wide distribution within social life, since trigeminal stimulation is used as a freshness effect. On the other hand, the simultaneous trigeminal stimulation in addition to the olfactory one is a feature that only occurs within a circumscribed group of odorants and thus facilitates the identification of these few substances compared to other, purely olfactory stimuli. However, it is then not possible to distinguish by which stimulus component the subject recognized the odorant, so that despite the good recognition rate, the suitability of peppermint for screening scent testing is clearly limited in this context.

The Sniffin’ sticks test for olfaction, which is well established in the clinical routine, distinguishes three different olfactory performances in the TDI score. Study results in COVID-19 patients indicate that especially the thresholds up to the perception of a scent are elevated. In contrast, suprathreshold tests for identification and discrimination did not show pathological results [40]. For the scratch test investigated here, it cannot be determined with certainty which olfactory performance is being tested exactly. It is most likely a combination of threshold and identification (possibly also discrimination, if row tests are taken into account). Thus, there is no specific testing of COVID-19 specific olfactory impairments.

The olfactory tests examined here do not allow a distinction to be made between an acute olfactory impairment (as is suspicious for the presence of a SARS-CoV-2 infection) and a pre-existing, chronic olfactory impairment (as is partly physiological as presbyosmia). This results in a proportion of false-positive tests (positive in the sense of a proven olfactory disorder) that are referred for further diagnostic testing without an underlying need. Thus, subjects with a pre-existing olfactory impairment would have to be preselected, as they are not suitable for screening by means of the scratch test.

## 5. Limitations

One of the main study limitations is attributable to the highly volatile nature of the pandemic. Although no DNA sequencing results are available, it can be assumed that most of the subjects in the study population were infected with the delta variant (B.1.617.2). The Omicron variant (initially B.1.1.529, later reclassified into BA.x lineages), first discovered in South Africa on 24 October 2021, has been the dominant variant since January 2022 compared to Delta, the most prevalent variant worldwide in December 2021 [42,43]. Omicron appears to cause less severe disease, which is likely to be mainly due to lower replication competence in the lung parenchyma compared to the bronchus include. Accordingly, the spectrum of symptoms is likely to differ from that observed in 2019 coronavirus disease caused by other SARS-CoV-2 strains (COVID-19) [44,45]. Despite the current shortage of published data comparing symptoms of the two variants, both animal models and the analysis of search databases show that anosmia seems to be much less pronounced in this virus variant [46,47,48]. The application of test procedures on the basis of clinical symptoms must be re-evaluated against this context, especially in such volatile situations.

## 6. Conclusions

Acute reduction of the sense of smell is described in many studies as an early and specific symptom of COVID-19 [49]. Therefore, a screening test of the sense of smell may be suitable to identify a- and oligosymptomatic infected individuals and to break chains of infection at an early stage. However, the use of scratch tests has many limitations. First, high selectivity in pre-test screening is needed to filter out subjects who are unsuitable for testing (e.g., in the presence of a language barrier or pre-existing hyposmia). Subsequently, various biases, such as environmental influences or geographic-cultural heterogeneities in scent sample familiarity, arise. Last, it must be explicitly emphasized that a preserved sense of smell does not exclude SARS-CoV-2 infection.

A possible application of the test could arise in the context of olfactory training as recommended after postviral and idiopathic olfactory impairments [50]. However, the usability and suitability for this need to be investigated in future studies.

## Figures and Tables

**Table 1:** Diagnostic test performance–comparison of different thresholds.

	Overall (*n* = 313)
**PCRRes**	
SARS-CoV-2 PCR positive	38 (12.1%)
SARS-CoV-2 PCR negative	275 (87.9%)
**Sex**	
male	138 (44.1%)
female	175 (55.9%)
**Age**	
Mean (SD)	31.9 (14.3)
Range	8.0–77.0
**hay fever/cold**	
No	223 (71.2%)
Yes	90 (28.8%)
**tested positive for SARS-CoV-2 last 2 month**	
No	272 (86.9%)
Yes	41 (13.1%)
**smoked last 2 h**	
No	268 (85.6%)
Yes	45 (14.4%)
**scent problems since longer time**	
No	247 (78.9%)
Yes (daily)	52 (16.6%)
Yes (occasionally)	14 (4.5%)
**subjective scent ability**	
very good	69 (22.0%)
good	143 (45.7%)
normal/ average	82 (26.2%)
bad	16 (5.1%)
very bad	1 (0.3%)
not specified	2 (0.6%)
**ability to inspire through nose**	
very good	36 (11.5%)
good	194 (62.0%)
normal/ average	34 (10.9%)
bad	45 (14.4%)
very bad	4 (1.3%)

**Table 2:** Diagnostic test performance–comparison of different thresholds.

	1 Out of 3	2 Out of 3	3 Out of 3	First Ticket Correct
**Negative Tests**	**265**	**177**	**76**	**163**
**True Test**	249	187	102	177
**Wrong Test**	64	125	210	136
**Sensitivity**	28.9 %	65.8 %	86.8 %	68.4 %
**Specificity**	86.5 %	59.6 %	25.8 %	54.9 %
**Accuracy**	79.6 %	60.4 %	33.2 %	56.5 %
**Prevalence**	4.8 %	4.8 %	4.8 %	4.8 %
**Positive Predictive Value**	22.9 %	18.4 %	13.9 %	17.3 %
**Negative Predictive Value**	89.8 %	92.7 %	93.4 %	92.6 %
**Post-test Disease Probability**	9.9 %	7.7 %	5.6 %	7.2 %
**Post-test Health Probability**	96.0 %	97.2 %	97.5 %	97.2 %
**Positive Likelihood Ratio**	2.15	1.63	1.17	1.52
**Negative Likelihood Ratio**	0.821	0.574	0.510	0.575

**Table 3:** Significance of the assay’s discriminative ability in reference to different subgroups (*: statistically significant with *p* ≤ 0.05).

		Correct Scenting	Incorrect Scenting	Two Tailed Fisher’s Exact Test
all participants	PCR +	31.6% (12)	68.4% (26)	***p*** = 0.0089 *
	PCR -	54.9% (151)	45.1 (124)	
Female	PCR +	46.2% (6)	53.8% (7)	*p* = 0.5649
	PCR -	56.8% (92)	43.2% (70)	
Male	PCR +	24% (6)	76% (19)	***p*** = 0.0141 *
	PCR -	52.2% (59)	47.8% (54)	
smoked last 2 h	PCR +	33.3% (2)	66.7% (4)	*p* = 0.4140
	PCR -	53.8% (21)	46.2% (18)	
cold/ hay fever	PCR +	13.3% (2)	86.7% (13)	***p*** = 0.0090 *
	PCR -	52% (39)	48% (36)	
Scenting sense: Good and very good	PCR +	100% (9)	0% (0)	***p*** = 0.0078 *
	PCR -	52.1% (25)	47.9% (23)	
tested positive for SARS-CoV-2 during the last 2 month	PCR +	35.7% (5)	64.3% (9)	*p* = 0.1147
	PCR -	63% (17)	37% (10)	

## Data Availability

The data presented in this study are available on request from the corresponding author.

## Supplementary Materials

The following supporting information can be downloaded at: https://www.mdpi.com/article/10.3390/life12070968/s1, Supplemental Material S1: Scan of the commercially available scratch test used in the study. Supplemental Material S2: Overview of the questionnaire and the possible answers and their scales. Supplemental Material S3: Distribution tree of test results in total number and percentages. Supplemental Material S4: Descriptive table of variables. Supplemental Material S5: corr plotTM: influence of environmental factors and their significance. Supplemental Material S6: overview of all subgroups and their percentage of correctly identified scents.
