# Supplementary material for: Anosmia Testing as Early Detection of SARS-CoV-2 Positivity; A Prospective Study under Screening Conditions
Source: Life (Basel). 2022 Jun 28;12(7):968. doi: 10.3390/life12070968 (PMC9319241; doi:10.3390/life12070968)
Supplement: Supplementary file 1 [file life-12-00968-s001.zip › life-1778144-supplementary.pdf]

inside

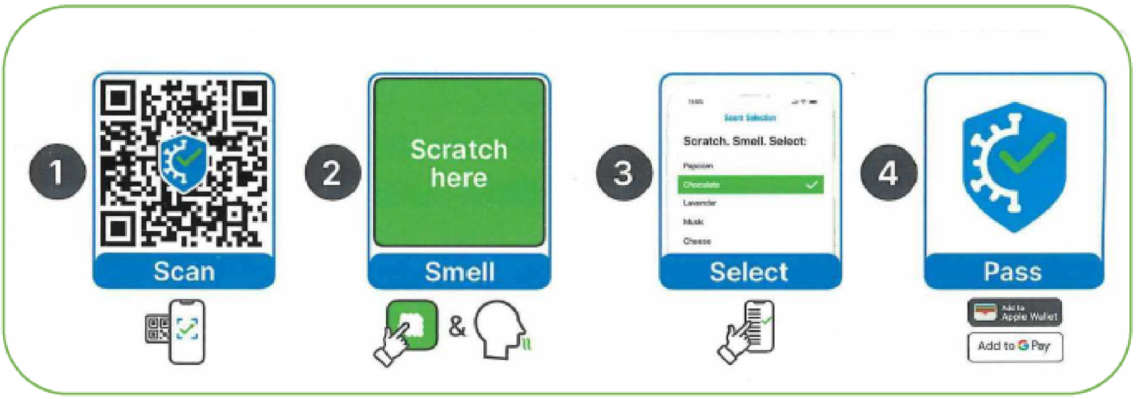

outside

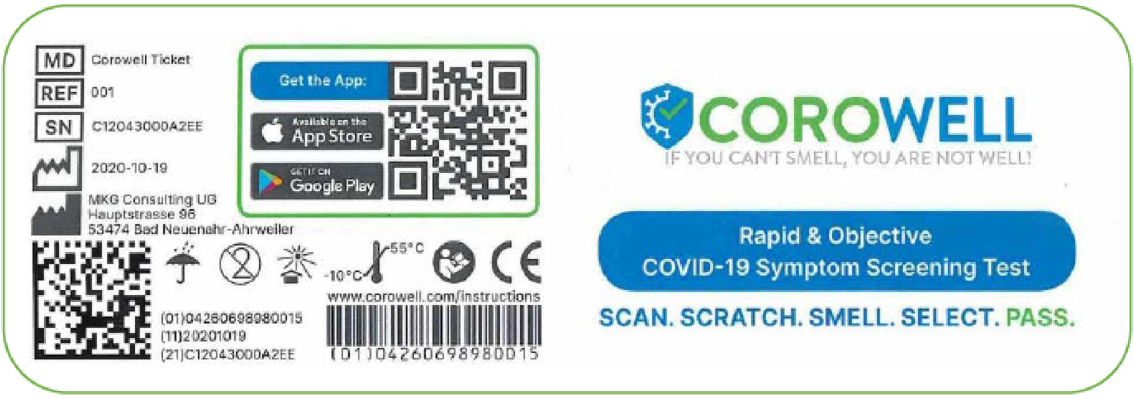

Supplemental Material S1: Scan of the commercially available scratch test used in the study.

gender	female; male; undefined
age	years
Already longer olfactory problems ?	No; yes (daily); yes (occasionally)
smoked during the last 2 hours	yes/ no
how do you value your scent ability in general?	five-level Likert item
Cold/ hay fever	yes/ no
How do you rate your ability breathing through your nose?	five-level Likert item
positive molecular genetic SARS-CoV-2 detection during the last 2 months	yes/ no

Supplemental Material S2: Overview of the questionnaire and the possible answers and their scales.

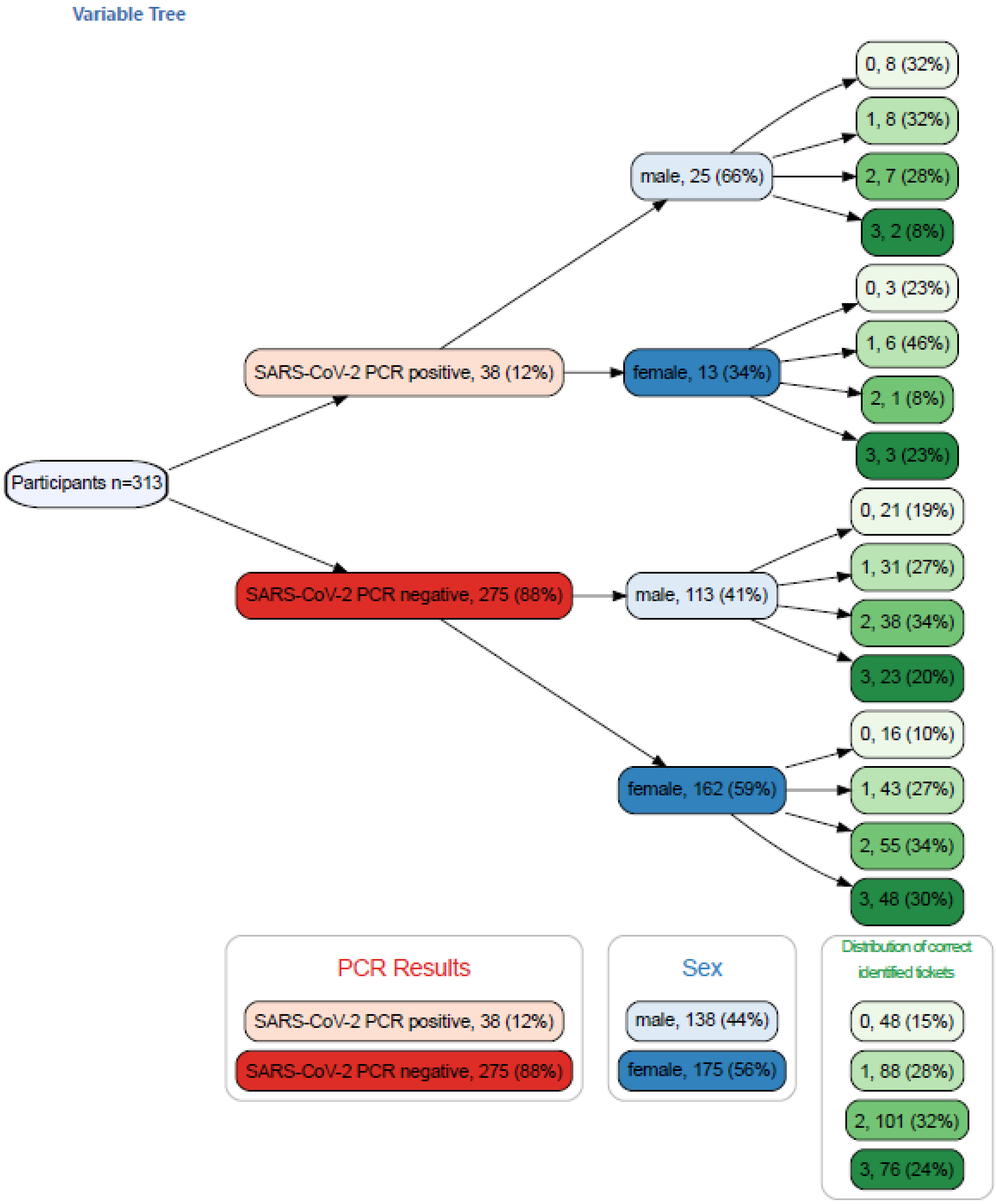

Supplemental Material S3: Distribution tree of test results in total number and percentages.

PCRRes	NEGATIVE	POSITIVE	
n=	275	38	
**Sex**			
male	113 (41.1%)	25 (65.8%)	
female	162 (58.9%)	13 (34.2%)	p=0.005
**Age**			
Mean (SD)	31.8 (14.4)	32.9 (13.3)	
Range	8.0 - 77.0	9.0 - 63.0	
**hay fever/ cold**			
No	200 (72.7%)	23 (60.5%)	
Yes	75 (27.3%)	15 (39.5%)	p=0.1287
**smoked last 2 hours**			
No	236 (85.8%)	32 (84.2%)	
Yes	39 (14.2%)	15 (39.5%)	p=0.8055
**scent problems since longer time**			
No	218 (79.3%)	29 (76.3%)	
Yes (daily)	45 (16.4%)	7 (18.4%)	
Yes (occasionally)	12 (4.4%)	2 (5.3%)	p=0.6736
**scent ability**			
very good	60 (21.8%)	9 (23.7%)	
good	128 (46.5%)	15 (39.5%)	
normal/ average	72 (26.2%)	10 (26.3%)	
bad	13 (4.7%)	3 (7.9%)	
very bad	1 (0.4%)	1 (2.6%)	
not specified	1 (0.4%)		p=0.3465
**ability to inspire through nose**			
very good	30 (10.9%)	6 (15.8%)	
good	175 (63.6%)	19 (50.0%)	
normal/ average	27 (9.8%)	7 (18.4%)	
bad	39 (14.2%)	6 (15.8%)	
very bad	4 (1.5%)		p=0.245
**tested positive for SARS-CoV-2 last 2 month**			
No	248 (90.2%)	24 (63.2%)	
Yes	27 (9.8%)	14 (36.8%)	p<0.001

Supplemental Material S4: Descriptive table Life 2022, 12, 0 14 of 16 of variables.

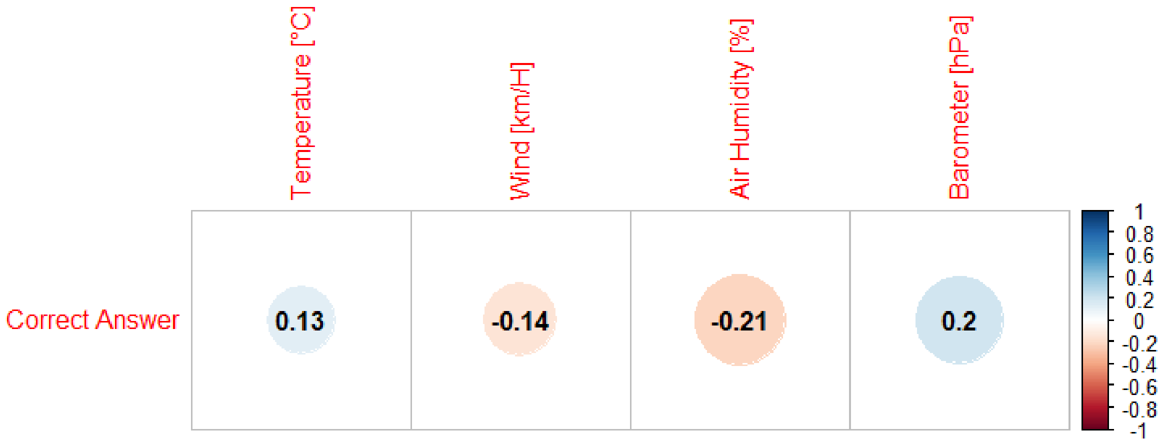

Supplemental Material S5: corr plotTM: influence of environmental factors and their significance.

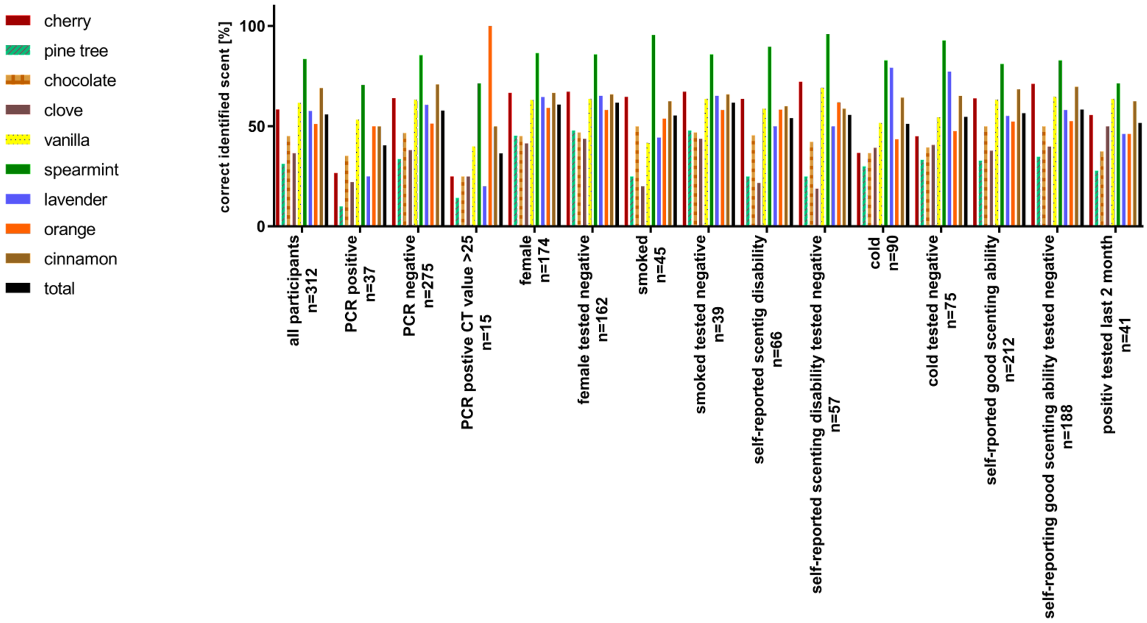

Supplemental Material S6: overview of all subgroups and their percentage of correctly identified scents.
